# Complete Genome Sequences of Two Novel Staphylococcus aureus Podoviruses of Potential Therapeutic Use, vB_SauP_phiAGO1.3 and vB_SauP_phiAGO1.9

**DOI:** 10.1128/genomeA.00048-18

**Published:** 2018-04-26

**Authors:** Agnieszka Gozdek, Aleksandra Głowacka-Rutkowska, Jan Gawor, Joanna Empel, Robert Gromadka, Małgorzata B. Łobocka

**Affiliations:** a Department of Microbial Biochemistry, Institute of Biochemistry and Biophysics of the Polish Academy of Sciences (IBB PAS), Warsaw, Poland; b Laboratory of DNA Sequencing and Oligonucleotide Synthesis, Institute of Biochemistry and Biophysics of the Polish Academy of Sciences, Warsaw, Poland; c National Medicines Institute (NMI), Warsaw, Poland; d Autonomous Department of Microbial Biology, Faculty of Agriculture and Biology, Warsaw University of Life Sciences, Warsaw, Poland

## Abstract

Here, we report the genome sequences of two Staphylococcus aureus phages belonging to the family Podoviridae and subfamily Picovirinae, vB_SauP_phiAGO1.3 and vB_SauP_phiAGO1.9, which were isolated from Warsaw sewage. Analysis of their genomes provides valuable information about the diversity of phages belonging to the genus Rosenblumvirus and their genes that undergo evolutionary adaptation to cells of different host strains.

## GENOME ANNOUNCEMENT

Phage therapy is a promising alternative to antibiotic therapy in the treatment of infections with antibiotic-resistant Staphylococcus aureus (1). The possibility of its use depends on the availability of phages against a given infecting strain. Lytic phages vB_SauP_phiAGO1.3 (phiAGO1.3) and vB_SauP_phiAGO1.9 (phiAGO1.9) were isolated in September 2009 from sewage at the Warsaw sewage farm Czajka using a standard procedure (2) with 14 different S. aureus strains. Both phages infected 11 of the same strains. A strain from the collection of the National Medicines Institute (NMI) (2064/05) was used as a phage propagator. It belongs to clonal complex 9 (CC9) and was isolated from cerebrospinal fluid of a patient diagnosed with invasive infection. Phages for DNA sequencing were precipitated using polyethylene glycol and NaCl from DNase- and RNase-treated cell lysates as described previously (3). DNA was extracted by using the SDS-proteinase K method (4) and sequenced at the Laboratory of Sequencing and Oligonucleotide Synthesis of the Institute of Biochemistry and Biophysics of the Polish Academy of Sciences (IBB PAS) using standard shotgun sequencing reagents and the Titanium 454 GS-FLX genome sequencer (Roche). The Lasergene version 9.1 software program (DNAStar, Inc.) was used for the sequence assembly, which resulted in 80 to 91× coverage. Ambiguous sequence regions were amplified with specific primers and resequenced using the ABI PRISM dye terminator cycle sequencing ready reaction kit with AmpliTaq DNA polymerase, FS (Perkin Elmer), and ABI 377 automated sequencers (Perkin Elmer). The correctness of the sequence assembly was verified by comparison of *in silico* and *in vivo* patterns of DNA digestion with PagI. Ends of virion DNA were identified by analyzing the PagI restriction pattern of phage DNA treated with BalI endonuclease, as described previously (5). Terminal repeat regions were resequenced by Sanger sequencing with virion DNA as a template and primers complementary to the nonredundant parts of the end regions (5′-GTTGCAACATTTCAGTATATGCTTG and 5′-GTTTTCGTTTTCTTCTTCGATTG). The genomes were annotated with the use of the Rapid Annotations using Subsystems Technology (RAST) server (6, 7).

The genomes of phiAGO1.3 and phiAGO1.9 consist of 17,603 and 17,637 bp, respectively, with an average GC content of 28.96%, which is lower than that of S. aureus (32.7%). Virion DNA molecules of both phages end with inverted terminal repeats (ITRs) that are perfect over the first 116 bp from each end, except for one mismatch, and less perfect until position 344 of the left ITR. The genomic sequences of phiAGO1.3 and phiAGO1.9 are nearly identical, with both phages encoding 20 proteins. Differences at the DNA level are limited to single mismatches in the region of the ITRs and the presence in open reading frame 20 (ORF20) in phiAGO1.9 of an additional 36-bp sequence repeat. Due to the insertion, the products of phiAGO1.3 and phiAGO1.9 ORF20 differ by 12 amino acid residues. At the genomic level, both phages are 91% identical to the staphylococcal podoviruses GRCS (8), BP39 (GenBank accession number KM366100), and SAP-2 (9) belonging to the Rosenblumvirus genus; they are also similar to other phages in this genus, although to a lesser extent. Significant differences concern proteins associated with phage-host interactions, namely, the minor tail fiber, the peptidoglycan-binding protein, amidase, and phage lysin, suggesting that phiAGO1.3 and phiAGO1.9 differ from their relatives in strain specificity.

### Accession number(s).

The genome sequences of S. aureus phages vB_SauP_phiAGO1.3 and vB_SauP_phiAGO1.9 were deposited in GenBank under the accession numbers MG766218 and MG766219, respectively.
